# Newborn weight change and predictors of underweight in the neonatal period in Guinea‐Bissau, Nepal, Pakistan and Uganda

**DOI:** 10.1111/mcn.13396

**Published:** 2022-07-12

**Authors:** Valerie J. Flaherman, Amy S. Ginsburg, Victoria Nankabirwa, Augusto Braima da Sa, Alvaro Medel‐Herrero, Eric Schaefer, Srijana Dongol, Akina Shrestha, Imran Nisar, Muddassir Altaf, Khushboo Liaquat, Benazir Baloch, Najeeb Rahman, Yasir Shafiq, Shabina Ariff, Fyezah Jehan, Susan B. Roberts

**Affiliations:** ^1^ Department of Pediatrics University of California San Francisco California USA; ^2^ Clinical Trials Center University of Washington Seattle Washington USA; ^3^ School of Public Health Makerere University Kampala Uganda; ^4^ International Partnership for Human Development Bissau Guinea‐Bissau; ^5^ Penn State College of Medicine Hershey Pennsylvania USA; ^6^ Kathmandu University School of Medical Sciences Dhulikhel Nepal; ^7^ Department of Paediatrics and Child Health Aga Khan University Karachi Pakistan; ^8^ The Gerald J and Dorothy R Friedman School of Nutrition Science and Policy Tufts University Boston Massachusetts USA

**Keywords:** growth, low‐ and middle‐income countries, newborn, underweight

## Abstract

In low‐ and middle‐income countries (LMIC), growth impairment is common; however, the trajectory of growth over the course of the first month has not been well characterised. To describe newborn growth trajectory and predictors of growth impairment, we assessed growth frequently over the first 30 days among infants born ≥2000 g in Guinea‐Bissau, Nepal, Pakistan and Uganda. In this cohort of 741 infants, the mean birth weight was 3036 ± 424 g. For 721 (98%) infants, weight loss occurred for a median of 2 days (interquartile range, 1–4) following birth until weight nadir was reached 5.9 ± 4.3% below birth weight. At 30 days of age, the mean weight was 3934 ± 592 g. The prevalence of being underweight at 30 days ranged from 5% in Uganda to 31% in Pakistan. Of those underweight at 30 days of age, 56 (59%) had not been low birth weight (LBW), and 48 (50%) had reached weight nadir subsequent to 4 days of age. Male sex (relative risk [RR] 2.73 [1.58, 3.57]), LBW (RR 6.41 [4.67, 8.81]), maternal primiparity (1.74 [1.20, 2.51]) and reaching weight nadir subsequent to 4 days of age (RR 5.03 [3.46, 7.31]) were highly predictive of being underweight at 30 days of age. In this LMIC cohort, country of birth, male sex, LBW and maternal primiparity increased the risk of impaired growth, as did the modifiable factor of delayed initiation of growth. Interventions tailored to infants with modifiable risk factors could reduce the burden of growth impairment in LMIC.

## Key messages

- In our study, 98% of infants initially lost weight after birth before beginning weight gain.
- Although most infants began weight gain by 2 days of age, those with delayed initiation of weight gain were more likely to be underweight at 30 days of age.
- Male sex, country of birth, low birth weight, maternal primiparity and delayed initiation of weight gain were predictors of being underweight at 30 days of age and of wasting at 30 days of age.

## Graphical abstract

In low‐ and middle‐income countries, growth impairment is common; however, the trajectory of growth over the course of the first month has not been well characterized. To describe newborn growth trajectory and predictors of growth impairment, we assessed growth frequently over the first 30 days among infants born ≥2000 grams (g) in Guinea‐Bissau, Nepal, Pakistan, and Uganda. In this cohort, country of birth, male sex, LBW and maternal primiparity increased the risk of impaired growth, as did the modifiable factor of delayed initiation of growth.

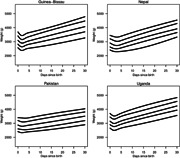

## 1 INTRODUCTION

Inadequate growth during infancy is common in low‐ and middle‐income countries (LMIC) and is associated with an increased risk of cognitive and immune impairment as well as susceptibility to infectious diseases (de Onis & Branca, 2016; Lu et al., 2016; Miller et al., 2016; UNICEF/WHO/World Bank Group, 2018). The first months after birth are critical for establishing healthy growth, particularly among newborns at risk due to economic or environmental factors hindering adequate nutrition (Abera et al., 2018; Admassu et al., 2018; Hui et al., 2010). Data from high‐income countries (HIC) show that during the newborn period, infants initially lose 5%–7% of their birth weight and then begin growth at about 3 days of age, increasing their weight by about 30% by 30 days of age (Bertini et al., 2015; Fonseca et al., 2015; Paul et al., 2016). Data from the World Health Organization (WHO) Multicentre Growth Reference Study, which enroled infants in both HIC and LMIC without health, environmental or economic risks to constrain growth, similarly demonstrates initial newborn weight loss followed by a gain of about 30% of birth weight by 30 days of age (de Onis et al., 2004).

Despite this robust normative data for weight change over the first 30 days in low‐risk infants, few studies have examined newborn weight change among at‐risk infants in LMIC, including those with health, environmental or economic risks for growth impairment. A cohort in western Nepal reported initial newborn weight loss of 7% of birth weight before the initiation of weight gain but followed infants only through 72 h of age (Gupta et al., 2018). Cohorts in Guinea‐Bissau, Uganda and Burkina Faso have described early infant growth of approximately 12%–17% per month but did not assess infants during the first week of life (Engebretsen et al., 2014; Jakobsen et al., 2008). No studies have examined growth from birth through the first month of life in at‐risk LMIC populations. Thus, we undertook a prospective cohort study to examine newborn growth in at‐risk LMIC populations and identify early predictors of inadequate growth at 30 days of age.

## 2 METHODS

At health facilities in Guinea‐Bissau, Nepal, Pakistan and Uganda between April 2019 and March 2020, we enroled 741 singleton infants who weighed ≥2000 g, a weight which was eligible for routine clinical care at all enroling sites. Additionally, infants were eligible if their mothers were ≥18 years old and intended to breastfeed for at least 6 months. The health facilities included Simoa Mendes Hospital in Bissau, Guinea‐Bissau, Bissora Hospital in Bissora, Guinea‐Bissau and village facilities and home births in Guinea‐Bissau; Dhulikhel Hospital in Dhulikhel, Nepal; Aga Khan University, Karimabad Hospital and Koohi Goth Health Center in Karachi, Pakistan; and Mukono Health Center in Mukono, Uganda and Kitebi Health Center and Kawala Health Center in Kampala, Uganda. We excluded infants with major congenital anomalies, danger signs, respiratory distress or maternal or infant contraindications to breastfeeding, but did not exclude infants with economic or environmental constraints on growth or specify gestational age parameters for study participation. We used a convenience sampling strategy for the selection of enrolment sites and infants. Sites in Guinea‐Bissau, Pakistan and Uganda had completed study activities before the onset of the COVID‐19 pandemic; the Nepal site had just completed enrolment at the time of the first COVID‐19 shutdown and was able to complete study activities.

Trained study staff recruited, screened and enroled mothers and infants and informed consent was obtained from the mother for herself and her infant. This study was approved by the UCSF Institutional Review Board, the Guinea‐Bissau National Committee on Ethics in Health (Comite Nacional de Etica na Saude), the Nepal Health Research Council, the Institutional Review Committee of Kathmandu University Teaching Hospital, the Ethical Review Committee at the Aga Khan University in Pakistan, the Higher Degrees, Research and Ethics Committee of Makerere University and the Uganda National Council of Science and Technology.

Using a standardised protocol, trained study staff obtained duplicate weights and lengths for naked infants at study visits, which were within 6 h of birth and at 1, 2, 3, 4, 5, 12 and 30 days of age, with a Seca 334 scale (Seca Inc.) accurate to ±5 g and stadiometer (Seca Inc and Pelstar, LLC); two additional measurements were taken if the initial two measurements varied by 15 g and 0.5 cm, respectively, for weight and length. We excluded weights obtained on Day 1 from analysis if they varied by 15% or more from birth weight and weights obtained on Days 2, 3 and 4 if they varied by 10% or more from the prior day's weight. Weights were excluded in this manner from seven infants on Day 1, three infants on Day 2, five infants on Day 3, five infants on Day 4 and four infants on Day 5. We did not exclude any weights obtained on Days 12 or 30 due to a lack of certainty regarding plausible weight change in those time intervals.

Infant dietary intake including breastfeeding and any supplementary feeding was assessed at these study visits using an instrument previously validated for breastfeeding infants in the first week of life in LMIC (Tylleskär et al., 2011). All enroled mothers were also surveyed regarding covariates related to enroled infant growth, including maternal age, educational attainment, marital status, parity, location of delivery, water source and type of toilet facility. All study visits occurred at the enrolment health facilities or during home visits as preferred by the participants. If necessary, participants were traced and located using provided contact information and maps. The study was strictly observational: the study team did not have access to data on changes in weight, did not provide health care to enroled infants and encouraged all mothers to access their usual sources of care after study enrolment. Referrals to medical care were made by the study team as needed. No direct care was provided by the study team, and ill infants were referred. Travel reimbursement was provided; no other incentives were provided.

Birth weight was defined as weight measured by trained study staff at <6 h of age. Low birth weight (LBW) was determined using the WHO definition of birth weight less than 2500 g. Underweight, stunting, and wasting were defined as weight‐for‐age *z*‐score (WAZ) <−2, length‐for‐age *z*‐score (LAZ) <−2 and weight‐for‐length *z*‐score (WLZ) <−2, respectively, and calculated using the WHO Anthro Survey Analyzer (World Health Organization, 2010, 2020) which was selected because reliable data on gestational age was not available for most of the cohort. Of note, this approach was unable to generate WLZ for lengths <45 cm, so WLZ was not used as a prespecified outcome. Our prespecified primary outcome was WAZ at 30 days of age because WAZ was expected to change more substantially over the first 30 days than LAZ.

Quantile regression methods appropriate for data with repeated measures were used to estimate 10th, 25th, 50th (median), 75th and 90th percentiles of weight (in g) as a function of time after birth separately for each country to depict weight changes during this period (Koenker, 2004). A restricted cubic spline with four degrees of freedom was used to generate nonlinear quantile curves, and the tuning parameter (*λ*) was set to 10 (Koenker et al., 1994).

To test associations with dichotomous outcomes, *χ* ^2^ and Student's *t*‐test were used for the bivariate analysis of dichotomous and normally distributed continuous variables, respectively; Wilcoxon rank sum test was used for bivariate analysis of continuous variables that were not normally distributed. We used Wald‐based confidence intervals to report the relative risk (RR) of dichotomous outcomes. Modified Poisson regression with robust standard errors was used to determine the relationship between baseline characteristics present at birth and dichotomous outcomes at 30 days of age while adjusting for potential confounders. All analyses were conducted in Stata/IC 16.0 (Stata Corp).

## 3 RESULTS

In this cohort, 321 infants were enroled in South Asian (SA) sites, Nepal and Pakistan and 420 in sub‐Saharan African (SSA) sites, Guinea‐Bissau and Uganda. All mothers were breastfeeding at enrolment, and only 3 (0.4%) reported having ceased breastfeeding at 30 days of age. Supplementary formula was reported on the day after birth and on Day 30, respectively, for 0 and 0 infants in Guinea‐Bissau, 1 (0.5%) and 1 (0.5%) infant in Uganda, 3 (3%) and 14 (17%) infants in Nepal, and 82 (37%) and 35 (16.6%) infants in Pakistan. Supplementary feeding other than formula, including milk, tea, soup, alcohol and honey, was reported on the day after birth and on Day 30, respectively, for 0 and 8 (4%) infants in Guinea‐Bissau; 97 (48%) and 34 (17%) infants in Uganda; 0 and 19 (24%) infants in Nepal; and for 45 (20%) and 7 (3%) infants in Pakistan. Overall, formula use was more common among LBW infants, occurring on the day after birth for 15 LBW infants (20%) compared to 73 (11%) non‐LBW infants (*p* = 0.03), and on Day 30 for 10 LBW infants (13%) compared to 39 (6%) non‐LBW infants (*p* = 0.02). Supplementary feeding other than formula did not differ between LBW and non‐LBW infants. Neonatal mortality occurred before 30 days of age for three infants (one each in Guinea‐Bissau, Pakistan and Uganda).

### 3.1 Birth characteristics

Baseline maternal characteristics such as education, marital status, household size and primiparity as well as infant characteristics such as weight and length differed by study site (Table 1). Overall mean birth weight in this cohort was 3036 ± 424 g (range 2000–4523 g) and was 203 g higher at SSA sites than at SA sites (*p* < 0.0005). Consistent with this finding, LBW was more common at SA sites compared to SSA sites (16% vs. 6.4%; *p* < 0.001). The cohort included 260 (49%) male infants, who weighed 88 g more at birth than females (*p* = 0.005).

**Table 1:** Birth characteristics, by country and region

	South Asia		Sub‐Saharan Africa	
Variables	Pakistan	Nepal	Uganda	Guinea‐Bissau
Infant characteristics at birth				
Male (%)	104 (47.3)	49 (48.5)	99 (48.3)	108 (50.2)
Birth weight, g (SD)	2908.9 (396.1)	2947 (404.3)	3157 (407.8)	3091.7 (434.2)
Birth length, cm (SD)	49.3 (1.7)	49.2 (1.3)	48.2 (2)	48.8 (2.2)
Low birth weight, *n* (%)	35 (16.4)	15 (15.3)	8 (4)	18 (8.9)
Caesarean delivery, *n* (%)	81 (36.8)	41 (41)	0 (0)	5 (2.3)
Maternal characteristics at infant birth				
Age, years (SD)	26.7 (4.8)	26.3 (4.5)	25.3 (4.9)	26.7 (5.7)
Weight, kg (SD)	63.3 (12.1)	64.6 (14.1)	62.5 (12.4)	62.4 (12.3)
Height, cm (SD)	157.9 (5.8)	155.3 (6.7)	157.6 (6.4)	159.2 (5.1)
Body mass index (SD)	25.3 (4.4)	26.4 (3.8)	24.8 (3.4)	24.6 (4.4)
Body mass index < 21, *n* (%)	37 (16.8)	5 (5.7)	20 (10.5)	51 (25.2)
Haemoglobin, g/dl (SD)	11.1 (1.3)	12.1 (1.1)	11.8 (1.6)	11.7 (1.8)
HIV, *n* (%)	1 (0.5)	0 (0)	6 (2.9)	0 (0)
Education (years) mean (SD)	8.1 (6.4)	14.2 (4)	9.4 (3.2)	5.9 (4.7)
Household size, *n (*SD)	7.6 (3.4)	6 (2.1)	4.3 (1.6)	9.1 (6.6)
Married, *n* (SD)	219 (100)	100 (100)	187 (92.6)	203 (99.5)
Primiparous, *n* (%)	73 (33.3)	58 (58)	67 (32.8)	65 (33.2)

Abbreviations: HIV, human immunodeficiency virus; *n*, number; SD, standard deviation.

### 3.2 Anthropometric changes over the first 30 days

Mean weight at 30 days of age was 3934 ± 592 g (range 2265–5585 g), which corresponded to a WAZ of −0.73 ± 1.1 (range −4.06–2.18). The mean birth length was 48.9 ± 1.9 cm (range 43.5–56 cm), which corresponded to a LAZ of −0.34 ± 1.0 (range −3.11 to 3.23). Mean weight change and mean length change over the first 30 days were 885.2 ± 397 g (29.5 ± 13.5% above birth weight), and 3.85 ± 1.70 cm (1.08 ± 0.3% above birth length), respectively. WLZ was not able to be calculated due to length below 45 cm for 15 infants on the day of birth, 6 infants on Day 12 and 3 infants on Day 30.

Weight change over the first 30 days varied substantially by country and ranged from 587 ± 316 g (20.8 ± 11.8% above birth weight) in Pakistan to 1078 ± 313 g (34.6 ± 10.9% above birth weight) in Uganda (Table 2). At 30 days of age, 96 (14.3%) cohort infants were underweight (defined as WAZ <−2), including 40 (41%) who had been LBW and 56 who (59%) had not; 58 (8.6%) were stunted (defined as LAZ <−2), including 20 (34%) who had been LBW and 38 (66%) who had not. Among infants who were underweight at 30 days, birth weight ranged from 2000 to 3720 g; among those who were not underweight at 30 days, birth weight ranged from 2360 to 4523 g. The overall prevalence of being underweight at 30 days of age varied between the countries and ranged from 5.0% among infants enroled in Uganda to 30.9% among infants enroled in Pakistan.

**Table 2:** Anthropometric changes over the first 30 days, by country and region

	All	South Asia (SA)			Sub‐Saharan Africa (SSA)			SA vs. SSA *p* value
		Pakistan	Nepal	Total	Uganda	Guinea‐Bissau	Total	
Change in weight from birth weight, g (%)								
Day 1 (Days 0–1)	−105.7 (−3.4)	−61.1 (−2.1)	−99.5 (−3.4)	−73.2 (−2.5)	−133.2 (−4.2)	−129.2 (−4.2)	−131.2 (−4.2)	<0.0001
Day 2 (Days 0–2)	−136.2 (−4.4)	−88.5 (−3)	−149.3 (−5.1)	−106.8 (−3.6)	−136.5 (−4.3)	−180.9 (−5.8%)	−158.7 (−5.1)	<0.0001
Day 3 (Days 0–3)	−102.3 (−3.3)	−75.4 (−2.5)	−171.6 (−5.8)	−104.8 (−3.5)	−80.3 (−2.6)	−120.8 (−3.9%)	−100.3 (−3.2)	0.308
Day 4 (Days 0–4)	−66.1 (−2.2)	−67.7 (−2.2)	−147.2 (−5)	−92.4 (−3.1)	−20.8 (−0.7)	−70.7 (−2.2)	−45.8 (−1.4)	<0.0001
Day 5 (Days 0–5)	−29.7 (−1)	−57.7 (−1.9)	−137.5 (−4.7)	−81.4 (−2.7)	35.7 (1.1)	−16.4 (−0.5)	9.8 (0.4)	<0.0001
Day 30 (Days 0–30)	885.2 (29.5)	587.5 (20.8)	949.1 (32.5)	670.9 (23.5)	1077.7 (34.6)	975.8 (32.1)	1027.7 (33.4)	<0.0001
Change in weight at nadir								
Day of weight nadir, median (IQR)	2 (1–4)	4 (2–5)	3 (2–4)	3 (2–5)	1 (1–2)	2 (1–2)	2 (1–2)	<0.0001
Loss from birth weight at nadir, g (%)	−178.9 (−5.9)	−131.3 (−4.5)	−228.7 (−7.8)	−161.9 (−5.5)	−168.5 (−5.4)	−215.2 (−6.9)	−191.9 (−6.2)	0.049
Change in length at 30 days of age								
Length change Day 0–30, cm (%)	3.9 (8)	2.8 (5.7)	5.3 (10.9)	3.3 (6.8)	5 (10.4)	3.4 (7)	4.2 (8.7)	<0.0001

Abbreviation: IQR, interquartile range.

**Figure 1:**
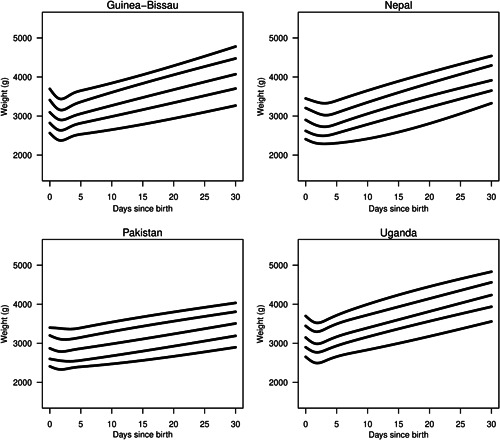
Infant weight over the first 30 days by country of birth depicted as the 10th, 25th, 50th (median), 75th and 90th percentiles of weight at each time point

Weight loss occurred before weight gain for 721 (98%) infants. Overall, infants lost 5.9 ± 4.3% of their birth weight before weight gain began at a median of 2 (interquartile range 1–4) days after birth (Figure 1). Infants who were underweight at 30 days of age had initiated weight gain at 4.3 ± 3.5 days of age while those who were not underweight at 30 days of age had initiated gain at 2.3 ± 1.5 days (*p* < 0.0005). Age at weight gain initiation was highly correlated with change in WAZ over the first 30 days; for each additional day of age at the time of weight gain initiation, WAZ at 30 days of age decreased by −0.20 (−0.18, −0.23). In particular, infants who reached weight nadir subsequent to 4 days of age were much more likely to be underweight at 30 days of age with a RR of 5.02 (3.46, 7.31) and were also much more likely to be wasted at 30 days of age with a RR of 4.65 (3.44, 6.28) (Table 3). Of note, at 30 days of age, eight (1.2%) infants weighed less than at birth, including five in Guinea‐Bissau and one in each of the other countries.

**Table 3:** Association of initial growth trajectory with weight‐for‐age *z*‐score (WAZ) <−2, length‐for‐age *z*‐score (LAZ) <−2 and weight‐for‐length *z*‐score (WLZ) < −2 at 30 days

	Underweight (WAZ < −2) at 30 days	Not underweight (WAZ ≥ −2) at 30 days	WAZ < −2 vs. WAZ ≥ −2; *p* value	Stunted (LAZ < −2) at 30 days	Not stunted (LAZ ≥ −2) at 30 days	LAZ < −2 vs. LAZ ≥ −2; *p* value	Wasted (WLZ < −2) at 30 days	Not wasted (WLZ ≥ −2) at 30 days	WLZ < −2 vs. WLZ ≥ −2; *p* value
Birth weight, g (SD)									
Birth weight	2599 (336)	3123 (379)	<0.00005	2629 (369)	3089 (398)	<0.00005	2736 (341)	3091 (408)	<0.00005
Change in weight from birth weight, g (% of birth weight)									
Day 1 (Days 0–1)	−73.9 (−2.7)	−111.2 (−3.5)	0.005	−104.1 (−3.9)	−105.8 (−3.4)	0.104	−71.9 (−2.6)	−111.7 (−3.6)	<0.0001
Day 2 (Days 0–2)	−110 (−4.1)	−138 (−4.4)	0.488	−117.6 (−4.5)	−135.4 (−4.3)	0.737	−110.2 (−3.9)	−138 (−4.4)	0.247
Day 3 (Days 0–3)	−104.7 (−3.8)	−95.4 (−3)	0.077	−89.7 (−3.4)	−96.8 (−3.1)	0.563	−119.3 (−4.2)	−92 (−2.9)	0.003
Day 4 (Days 0–4)	−105.7 (−3.8)	−51.8 (−1.6)	*p* < 0.0001	−43.2 (−1.6)	−60.2 (−1.9)	0.620	−119.9 (−4.3)	−47.6 (−1.5)	<0.0001
Day 5 (Days 0–5)	−101 (−3.6)	−7.4 (−0.2)	0.0001	−12.4 (−0.4)	−20.3 (−0.6)	0.745	−112.3 (−4)	−3 (0)	<0.0001
Day 30 (Days 0–30)	414.3 (17.2)	963.7 (31.5)	0.0001	857.4 (33)	887.9 (29.1)	0.033	383.1 (15.2)	974.6 (32)	<0.0001
Characteristics of weight nadir									
Day of weight nadir, median (IQR)	5 (2–5)	2 (1–3)	0.0001	2 (1–2)	2 (1–4)	0.013	5 (3–5)	2 (1–3)	<0.0001
Weight lost at nadir, grams (% of birth weight)	−161.4 (−5.9)	−175.4 (−5.6)	0.404	−154.3 (−5.9)	−174.3 (−5.6)	0.569	−169 (−6)	−173.2 (−5.6)	0.254
Linear growth									
Length change Day 0–30, centimeters (% of birth weight)	3 (6.3)	4 (8.2)	0.0001	3 (6.7)	3.9 (8.1)	0.006	3.2 (6.5)	4 (8.2)	<0.0001

Abbreviation: IQR, interquartile range.

### 3.3 Characteristics predictive of growth attainment at 30 days

Being underweight at 30 days of age was associated with birth in the SA region (RR 3.89 [2.56, 5.91]), male sex (RR 2.37 [1.58, 3.57]), LBW (RR 6.56 [4.77, 9.04]) and maternal primiparity (RR 1.64 [1.12, 2.39]). In a multivariable analysis examining the relationship between being underweight at 30 days and country and sex while adjusting for birth weight, maternal age and maternal primiparity, the RRs for Guinea‐Bissau, Nepal and Pakistan compared to Uganda were 1.08 (0.51, 2.25), 0.87 (0.36, 2.15) and 2.76 (1.53, 5.02), respectively, with male sex and maternal primiparity continuing as strong predictors with RRs of 2.30 (1.59, 3.33) and 1.91 (1.35, 2.72), respectively (Table 4). A similar model which also adjusted for characteristics occurring subsequent to birth demonstrated a RR of 1.31 (1.21, 1.43) for each additional day to weight nadir and a RR of 1.19 (0.78, 1.80) for supplementation with formula on the day after birth. While adjusting for the same covariates, multivariable analysis examining the outcome of stunting at 30 days of age showed that male sex, time to weight nadir, and low birth weight remained strong predictors of growth, while primiparity and formula supplementation were not.

**Table 4:** Association between birth characteristics and being underweight at 30 days of age, with and without adjustment for time to weight nadir and supplementation

	Weight‐for‐age *z*‐score <−2 at 30 days of age (%)	Weight‐for‐age *z*‐score ≥−2 at 30 days of age (%)	*p* value	Relative risk of multivariable model adjusting for site	Relative risk of multivariable model adjusting for site, time to weight nadir and method of feeding
Country			<0.0005	–	
Guinea‐Bissau	17 (8.3)	187 (91.7)		1.08 (0.51, 2.25)	0.90 (0.44, 1.85)
Nepal	6 (9.7)	56 (90.3)		0.87 (0.36, 2.15)	0.70 (0.31, 1.57)
Pakistan	63 (30.9)	141 (69.1)		2.77 (1.53, 5.02)	2.13 (1.17, 3.86)
Uganda	10 (5.0)	191 (95.0)		Ref	Ref
Infant characteristics at birth					
Male, *n* (%)	67 (69.8)	264 (45.9)	<0.0001	2.30 (1.59, 3.33)	2.41 (1.69, 3.43)
Weight, g (SD)	2599 (336)	3123 (379)	<0.0001	0.99 (0.99, 0.99)	0.99 (0.99, 0.99)
Low birth weight, *n* (%)	38 (40.9)	24 (4.3)	<0.0001	–	
Maternal characteristics at infant's birth					
Primiparity, *n* (%)	43 (46.7)	188 (33)	0.010	1.91 (1.35, 2.72)	1.41 (0.96, 2.07)
Parity, median (IQR)	2 (1–2)	2 (1–3)	<0.0001	–	–
Age, years (SD)	27.3 (5.3)	26.2 (5.1)	0.052	1.05 (1.01, 1.08)	1.01 (0.97, 1.05)
Weight, km (SD)	64.2 (12.4)	62.9 (12.7)	0.373	–	–
Height, cm (SD)	157.3 (6.6)	158.3 (5.8)	0.136	–	–
Body mass index	25.8 (4.3)	24.9 (4)	0.040	–	–
Body mass index <21, *n* (%)	10 (10.5)	95 (16.9)	0.118	–	–
Haemoglobin, g/dl (SD)	11.5 (1.5)	11.6 (1.6)	0.373	–	–
Maternal education, years (SD)	9.6 (6)	8.4 (5.2)	0.034	–	–
Household size, *n* (SD)	7 (3.3)	7.1 (4.8)	0.883	–	–
Married, *n* (%)	95 (99)	556 (97.4)	0.348	–	–
Infant characteristics after birth				–	
Age at weight nadir (days) (SD)	4.28 (3.5)	2.29 (1.54)	<0.00005	–	1.31 (1.21, 1.43)
Supplementary feeding on day after birth, *n* (SD)	53 (55)	170 (29)	<0.0005	–	1.19 (0.78, 1.80)

Abbreviations: IQR, interquartile range; *n*, number; SD, standard deviation.

## 4 DISCUSSION

In this cohort of healthy breastfed infants born in LMIC without respiratory distress or congenital anomaly, the majority of infants who were underweight at 30 days of age had not been low birth weight but rather, had experienced poor initial weight gain. A more pronounced and extended period of initial weight loss before the initiation of weight gain was significantly associated with reduced growth attainment at 30 days of age, suggesting that efforts to promote adequate nutrition in the first few days after birth may reduce the risk of growth impairment. Male infants appear to be particularly vulnerable and were more than twice as likely to be underweight at 30 days of age as defined by the WHO Child Growth Standards. In our study, the risk of being underweight at 30 days of age varied substantially by country. Taken together, results from this study suggest that it may be feasible to identify apparently healthy newborns at higher risk of subsequent growth impairment and develop tailored interventions to improve outcomes.

Our findings are consistent with recent literature demonstrating the persistent, strong association of LBW with being underweight throughout early infancy in LMIC and the difficulty establishing healthy growth for this vulnerable LBW population (Benjamin‐Chung et al., 2020; Mertens et al., 2020). Male sex, birth in South Asia and maternal primiparity, identified as associated with being underweight at 30 days of age in our cohort, have also been flagged as risk factors in recent studies (Thurstans et al., 2020; Victora et al., 2021). Notably, the initial period of weight loss in the first few days after birth was shorter and less pronounced for our cohort than previously reported in high‐income countries (Bertini et al., 2015; Chantry et al., 2014; Flaherman et al., 2015; Fonseca et al., 2015; Paul et al., 2016). This difference might be attributable to any number of factors that influence early weight loss, including intrauterine growth retardation, use of intrapartum fluids, the timing of the onset of copious milk production or supplementary feeding practices. Of further note, the degree of initial weight loss in our study was more pronounced than WHO Child Growth Standards (World Health Organization, 2022) which may be attributable to the presence of suboptimal conditions for weight gain in the neonatal period in these study sites.

Importantly, the slower postnatal weight gain we identified in Nepal and Pakistan appears to have compounded weight disparities present at birth. Factors contributing to differences in birth weight and postnatal weight gain by location could include antenatal care practices, constitutional characteristics and early feeding behaviours. Further research is needed to understand the aetiology of inadequate prenatal and postnatal growth in LMIC.

Our study has several limitations. First, validated assessment of gestational age was not available at most of our sites, and caesarean births were rare, so we are unable to report how newborn growth varied by gestational age or delivery method. Since 10.6% of our cohort newborns were LBW, it is likely that the proportion of preterm infants was not insignificant. This is an important limitation because preterm birth influences supplementary feeding practices, time to weight nadir and growth trajectory in the first 30 days (Poulimeneas et al., 2021; Villar et al., 2015). However, since we included only infants born ≥2000 g, it is unlikely that our cohort contained any very or extremely preterm infants. Second, our study used convenience sampling to enrol 741 infants in four countries and may therefore not represent infant growth in any given country or region, or worldwide. Third, while all infants were breastfeeding at enrolment, we did not collect detailed breastfeeding data and are not able to report on the role of latch score or duration and frequency of breastfeeding in this cohort of infants. Fourth, although we used a carefully standardized method for weight measurement, 24 (0.01%) of the measured weights were excluded due to implausibility. Additional measurement errors likely occurred without generating implausible values, and this may impact the validity of our results. Of note, however, measurement error tends to bias results to the null, while many of our identified effect size estimates were large, suggesting that the study's conclusions are likely sound despite this limitation (Sterne et al., 2019).

Our study was able to examine the impact of these variables day‐to‐day in the first days after birth, and our results demonstrate that expeditiously initiating healthy weight gain in the first few days after birth is a crucial component of preventing newborns from being underweight and may be especially important for male infants and those born in SA. Interventions such as early initiation of breastfeeding and early feeding of expressed milk are effective for promoting healthy growth, and breastfeeding counselling and education support packages including early initiation of breastfeeding have been shown to be effective for improving infant growth in LMIC (Ahmadi et al., 2016; Froozani et al., 1999; Rana et al., 2021). Studying early growth patterns among LMIC infants without exclusion of those born with economic and social barriers to growth may allow the development of interventions tailored to those at the highest risk. Actively supporting early growth might reduce growth faltering and its concomitant impact on health for at‐risk infants in LMIC.

## AUTHOR CONTRIBUTIONS

Valerie J. Flaherman and Susan B. Roberts conceptualised and designed this study. Victoria Nankabirwa, Augusto Braima da Sa, Srijana Dongol, Akina Shrestha, Imran Nisar, Muddassir Altaf, Khushboo Liaquat, Benazir Baloch, Najeeb Rahman, Yasir Shafiq, Shabina Ariff and Fyezah Jehan completed the research. Alvaro Medel Herrero and Eric Schaefer analysed the data, Valerie Flaherman had primary responsibility for writing the manuscript and all authors have read and approved the final manuscript.

## CONFLICT OF INTEREST

All authors disclose support from the Bill & Melinda Gates Foundation which funded this research. Dr. Roberts also discloses funding from Danone, S.A., for unrelated work.

## Data Availability

Deidentified, individual‐level data on variables listed in tables will be shared with other researchers after approval of the proposed research question(s) and a signed data use agreement.
